# Supplementary material for: Coordination of siderophore gene expression among clonal cells of the bacterium Pseudomonas aeruginosa
Source: Commun Biol. 2022 Jun 6;5:545. doi: 10.1038/s42003-022-03493-8 (PMC9170778; doi:10.1038/s42003-022-03493-8)
Supplement: Supplementary file 2 — Supplementary Information [file 42003_2022_3493_MOESM2_ESM.pdf]

Coordination of siderophore gene expression among clonal cells of the bacterium *Pseudomonas aeruginosa*

Subham Mridha[1], Rolf Kümmerli[1]

[1]Department of Quantitative Biomedicine, University of Zürich, Winterthurerstrasse 190, 8057 Zürich, Switzerland

These supplementary materials contain:

- 10 supplementary Figures
- 4 supplementary Tables

SUPPLEMENTARY FIGURES

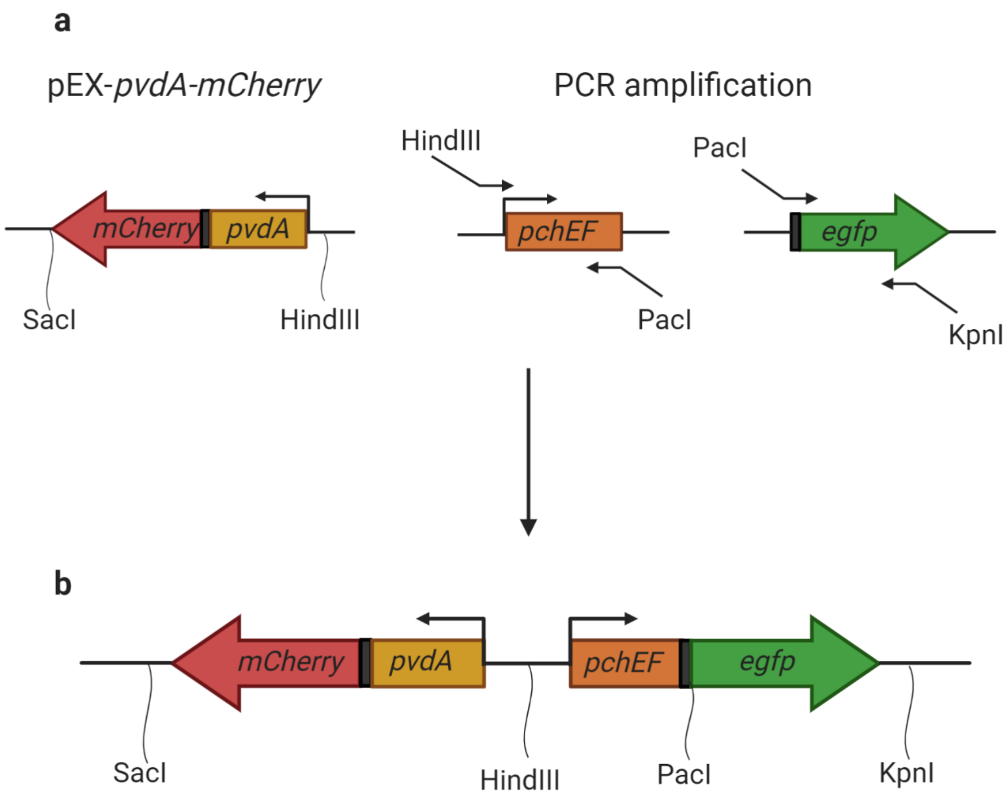

Figure S1. Double fluorescent gene reporter scaffold type 1 used for strain PAO1*pvdA*::*mcherry–pchEF*::*egfp*. (a) The promoters for the pyochelin synthesis genes *pchEF* and the fluorescent gene marker e*gfp* were PCR amplified using primers denoted by arrows with unique restriction enzyme sites. The vector pEX-*pvdA-mCherry* containing the promotor for the pyoverdine synthesis gene *pvdA* fused to the *mCherry* gene was digested at the denoted unique restriction enzyme sites. **(b)** Subsequently, the gene fragments were ligated at the specific restriction enzyme sites and integrated into the pUC18-miniTn7-Gm vector. Ribosomal binding sites are shown as dark brown rectangles at the start of the fluorescent gene markers *egfp* and *mCherry*.

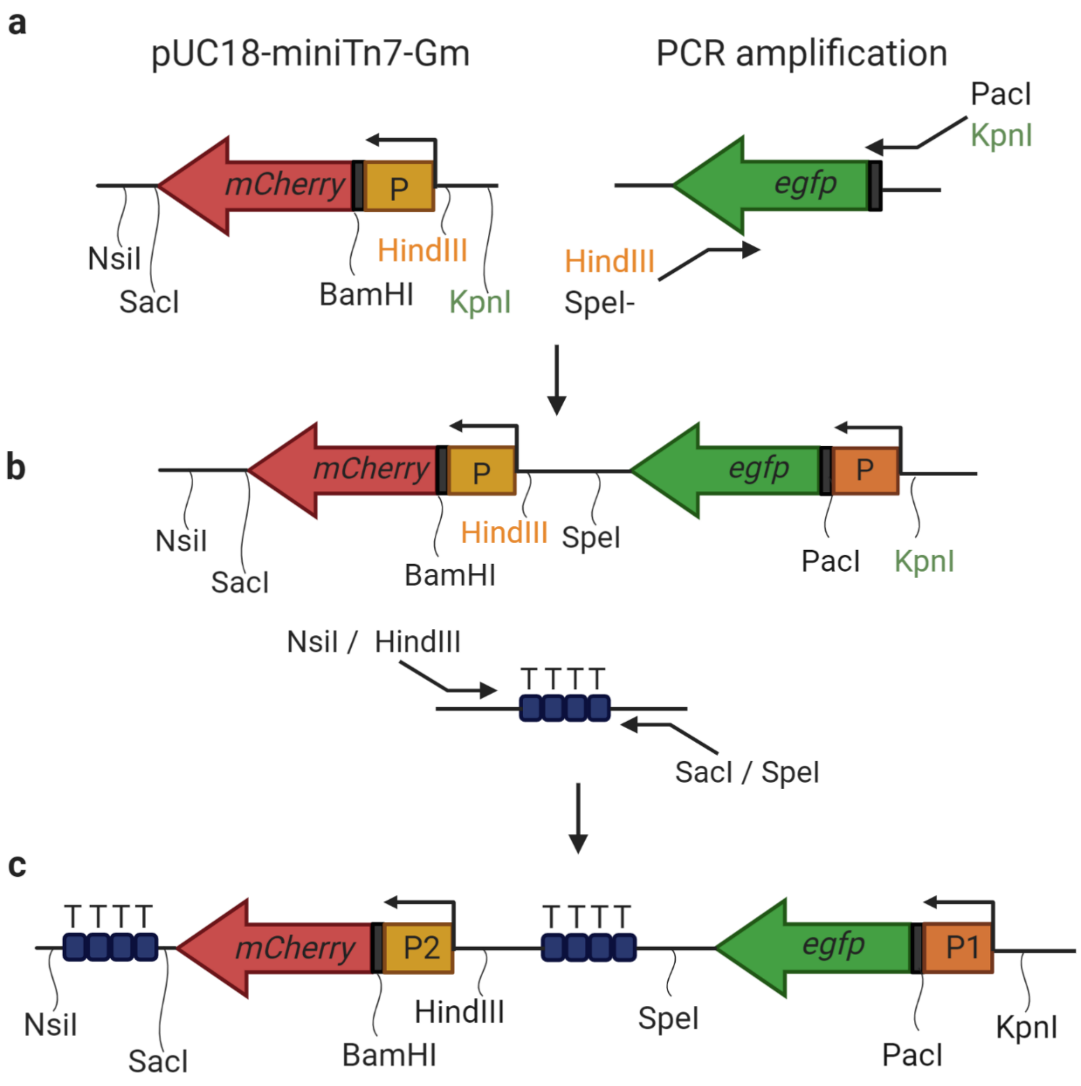

Figure S2. Double fluorescent gene reporter scaffold type 2 used for the strains PAO1*pchEF*::*mcherry–rpsL*::*egfp* and PAO1*pvdA*::*mcherry–rpsL*::*egfp*. (a)The fluorescent gene marker *egfp* was PCR amplified using the primers denoted by arrows with unique restriction enzyme sites. The PCR product contained an empty promoter region between the restriction enzyme sites PacI and KpnI. **(b)** Subsequently, the gene fragment was ligated at the specific restriction sites HindIII and KpnI (denoted by colour code of respective restriction enzyme) of the pUC18 miniTn7 Gm – *mCherry* vector plasmid containing an empty promoter site fused to *mCherry* . Four rho-independent terminators denoted by T (deep blue boxes) were also PCR amplified using two pairs of primers containing unique restrictions sites NsiI and SacI, and HindIII and SpeI. **(c)** Promoter sites are denoted as P1 (fused to *egfp*) and P2 (fused to *mCherry*). Promoter regions of the genes of interest were added at the sites P1 (fused to *egfp*) and P2 (fused to *mCherry*) using restriction enzyme sites KpnI and PacI or HindIII and BamHI, respectively. Promoter interference was eliminated through the ligation of terminator sites between two promoter fusions at the specific restriction sites HindIII and SpeI, and NsiI and SacI. Ribosomal binding sites are shown as dark brown rectangles at the start of the fluorescent gene markers *egfp* and *mCherry*.

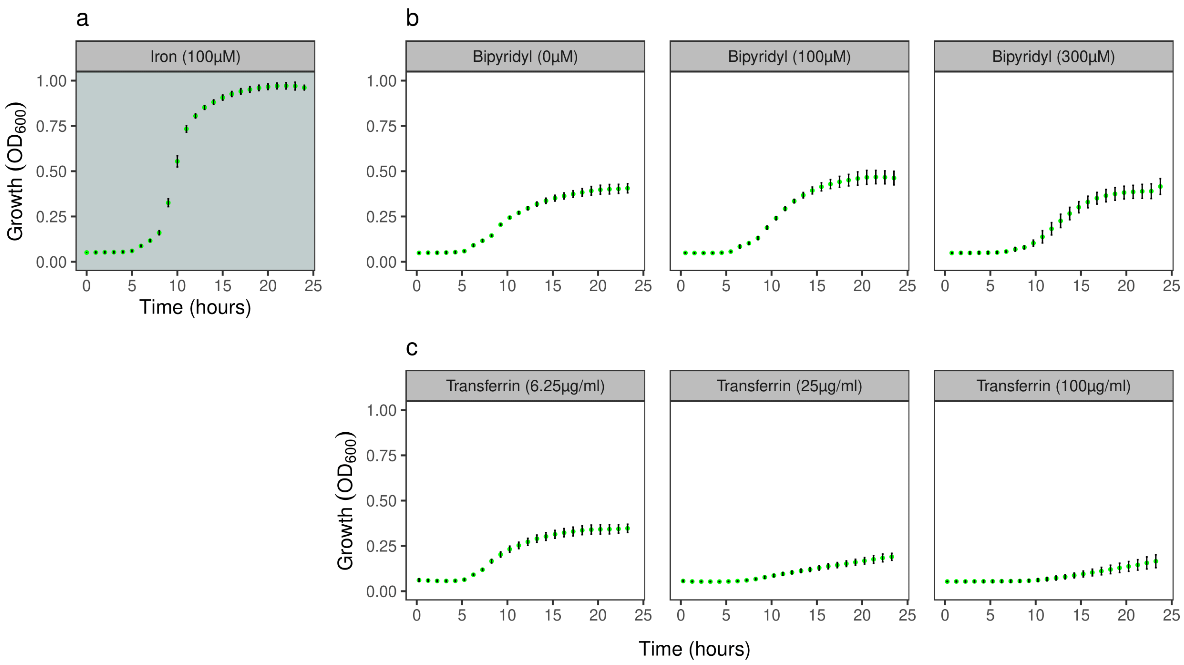

Figure S3. Population level growth kinetics. The panels show temporal dynamics of bacterial growth across a range of CAA media differing in their level of iron limitations. Values and error bars represent the mean growth and standard deviation across 24 replicates, in **(a)** iron-replete CAA medium (100 µM $FeCl_3$); **(b)** CAA media with increasing concentrations of the iron chelator bipyridyl; **(c)** CAA media with increasing concentrations of the iron chelator apo-transferrin. The bacterial growth is maximized in iron-replete CAA medium, and decreases with the addition of iron chelators. Note that we combined the data from three PAO1 strains (PAO1 wildtype and the two single gene reporter strains PAO1*pchEF*:*mcherry* and PAO1*pvdA*:*mcherry*) as there was no significant differences in growth between them.

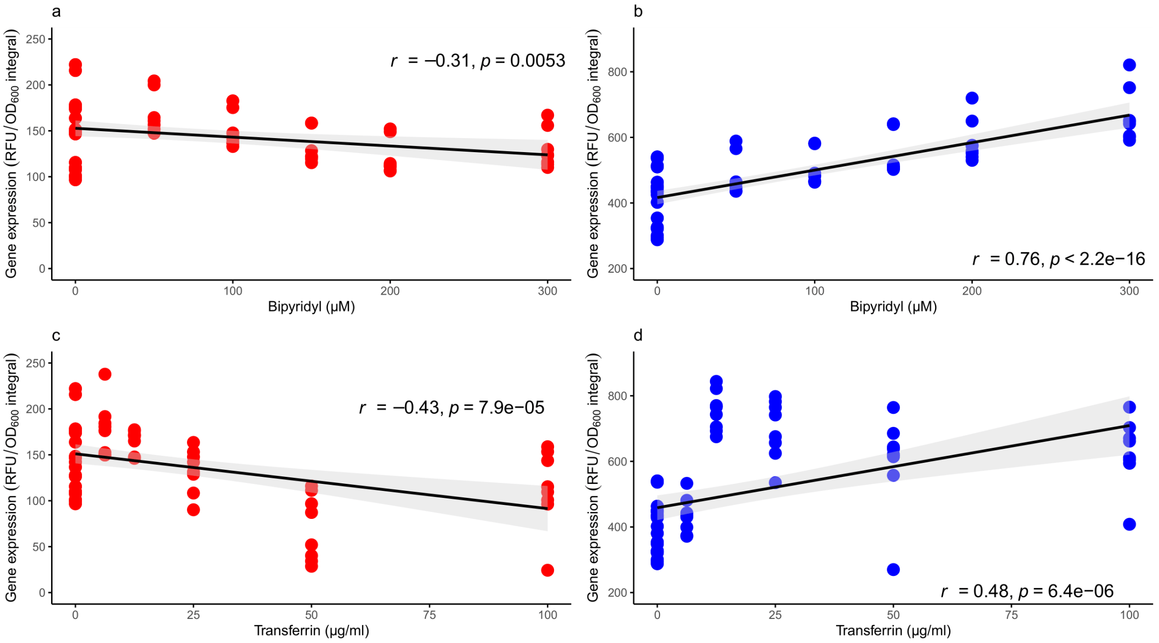

Figure S4. Correlations between siderophore gene expression and chelator concentration. The panels show siderophore reporter gene expression (red: pyochelin PAO1*pchEF*:*mcherry*; blue: pyoverdine PAO1*pvdA*:*mcherry*) measured as normalized integral (area under the fluorescence signal curve divided by the growth OD600 integral) over a growth period of 24 hours in batch cultures. Gene expression was measured as RFU (relative fluorescence unit) in CAA media with increasing concentrations of the iron chelators bipyridyl **(a+b)** and apo-transferrin **(c+d)**. Pyochelin gene expression **(a+c)** correlated negatively with chelator concentration, meaning that populations invested more into pyochelin at lower levels of iron limitation. In contrast, pyoverdine gene expression **(b+d)** correlated positively with chelator concentration, meaning that populations invested more into pyoverdine at higher levels of iron limitation. Black lines and shadings indicate significant trendlines and 95% confidence intervals, respectively. The corresponding Pearson correlation coefficient *r* and *p*-value are provided in each panel.

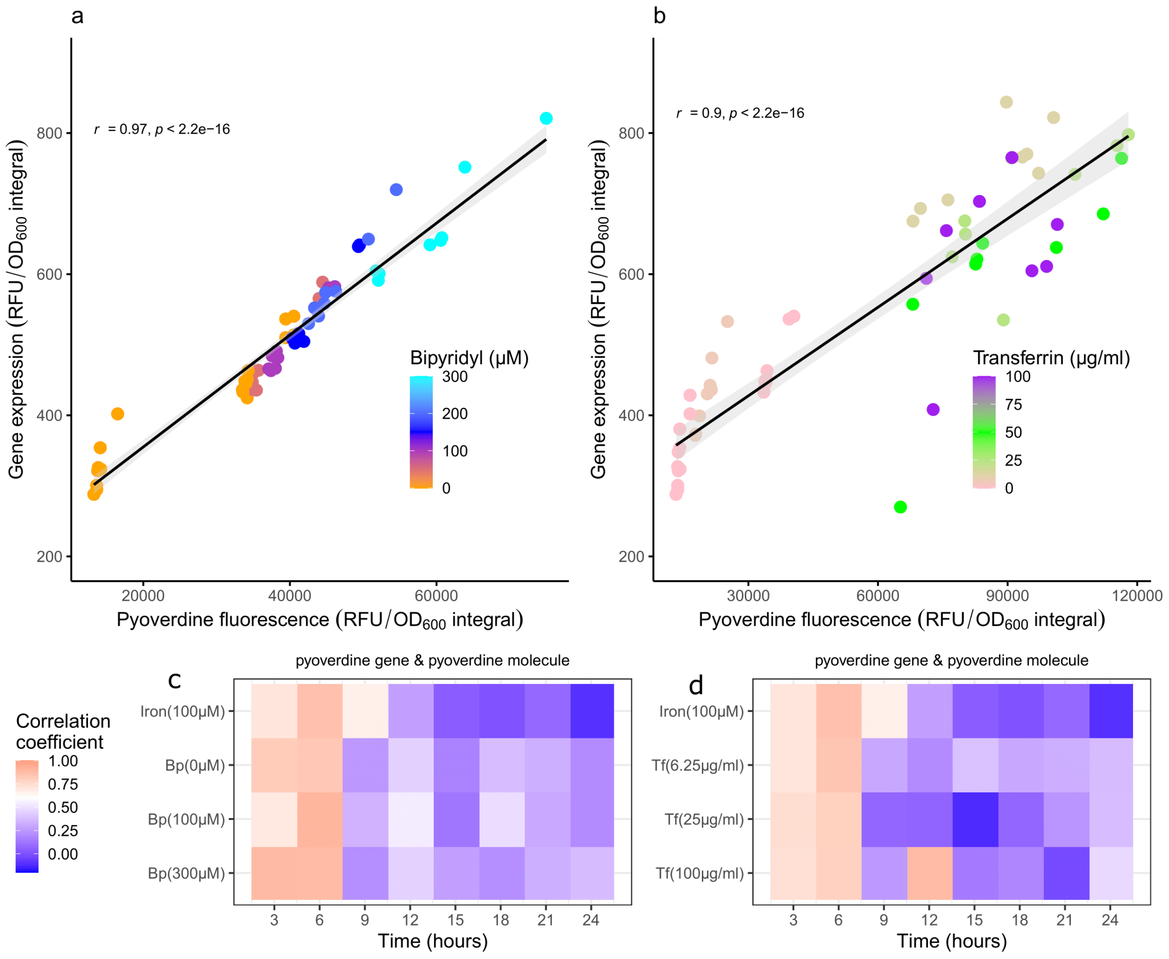

Figure S5. Correlation between pyoverdine gene expression and pyoverdine molecule autofluorescence. At the batch culture level **(a+b),** pyoverdine gene expression and natural pyoverdine autofluorescence were measured as normalized integral (area under the fluorescence signal curve divided by the growth OD600 integral) over a 24 hours growth cycle. The single reporter strain PAO1*pvdA*:*mcherry* was used and grown in CAA media supplemented with increasing concentrations of the iron chelators bipyridyl **(a)** and apo-transferrin **(b)**. Black lines and shadings indicate significant trendlines and 95% confidence intervals, respectively, between pyoverdine gene expression and molecule fluorescence. The corresponding Pearson correlation coefficient *r* and *p*-value are provided in each panel. At the single cell level **(c+d)**, pyoverdine gene expression and natural pyoverdine autofluorescence were measured and correlated across cells in three-hours intervals (as for the main experiment). Here, the double reporter strain PAO1*pvdA*::*mcherry–pchEF*::*egfp* was used and grown in CAA media supplemented with increasing concentrations of the iron chelators bipyridyl **(c)** and apo-transferrin **(d)**. The Pearson correlation coefficients are shown as heatmaps, and reveal strong positive correlations between pyoverdine gene expression and actual molecule production at early time points (up to 6 hours). At later time points, correlations become weaker. This is expected as pyoverdine is secreted and taken up by other cells. Thus, at later time points, the pyoverdine autofluorescence of a cell is the sum of pyoverdine production and uptake. Taken together, the correlation analyses show that gene expression is a good proxy for siderophore production.

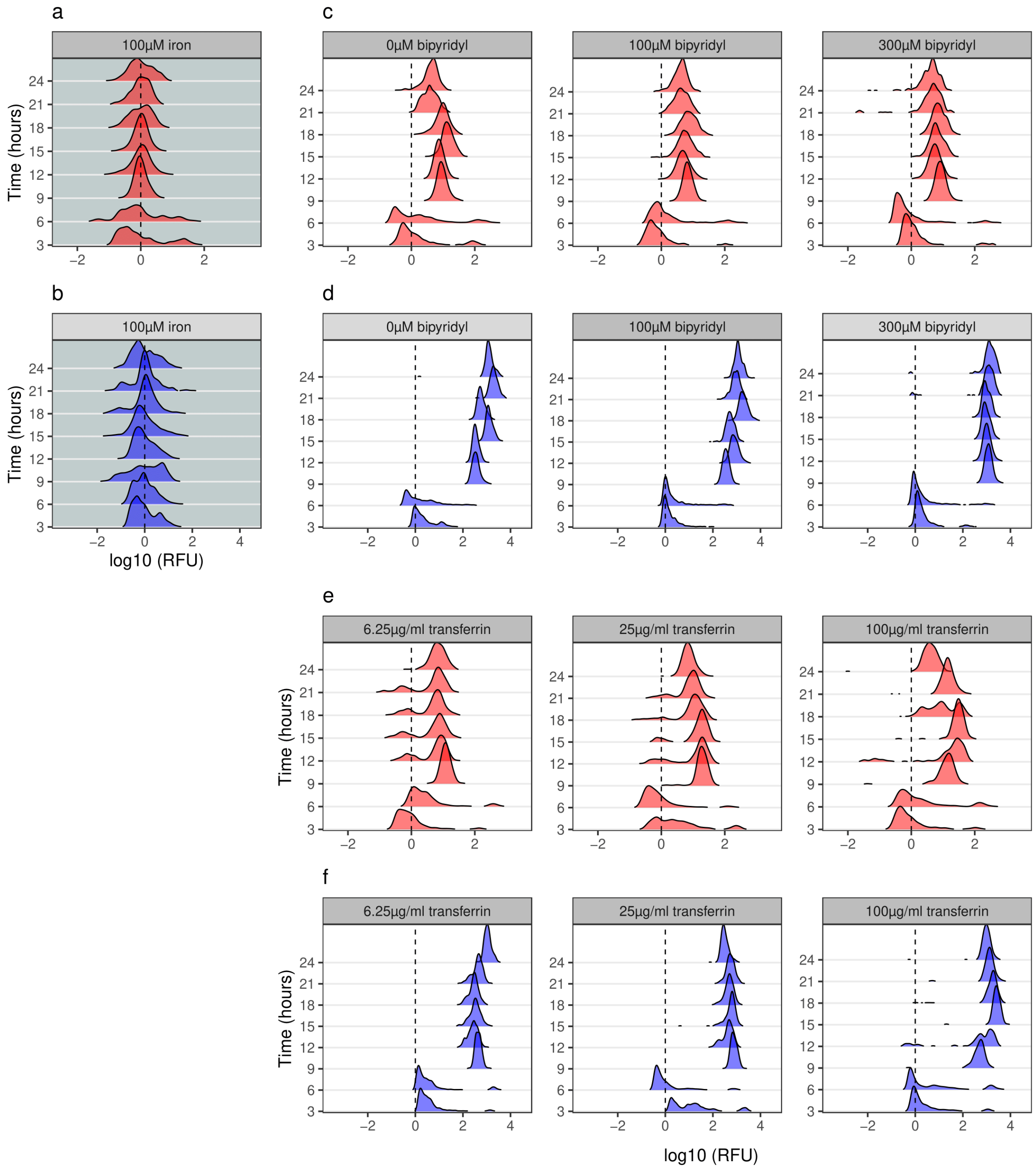

Figure S6. Single-cell siderophore gene expression shown as density plots across time and media. Single-cell siderophore gene expression shown as density plots for pyochelin (red: *pchEF*) and pyoverdine (blue: *pvdA)*, measured with the double reporter strain PAO1*pvdA*::*mcherry–pchEF*::*egfp* across a range of CAA media differing in their levels of iron limitations. The density plots represent the frequency distribution of log-transformed fluorescence values. **(a+b)** Iron-replete CAA medium (100 µM $FeCl_3$); **(c+d)** CAA media with increasing concentration of the iron chelator bipyridyl; **(e+f)** CAA media with increasing concentration of the iron chelator apo-transferrin. The black dashed line at 0 represents the background level of gene expression. Relative fluorescence units (RFU) of the reporter proteins represent the gene expression intensity of single cells.

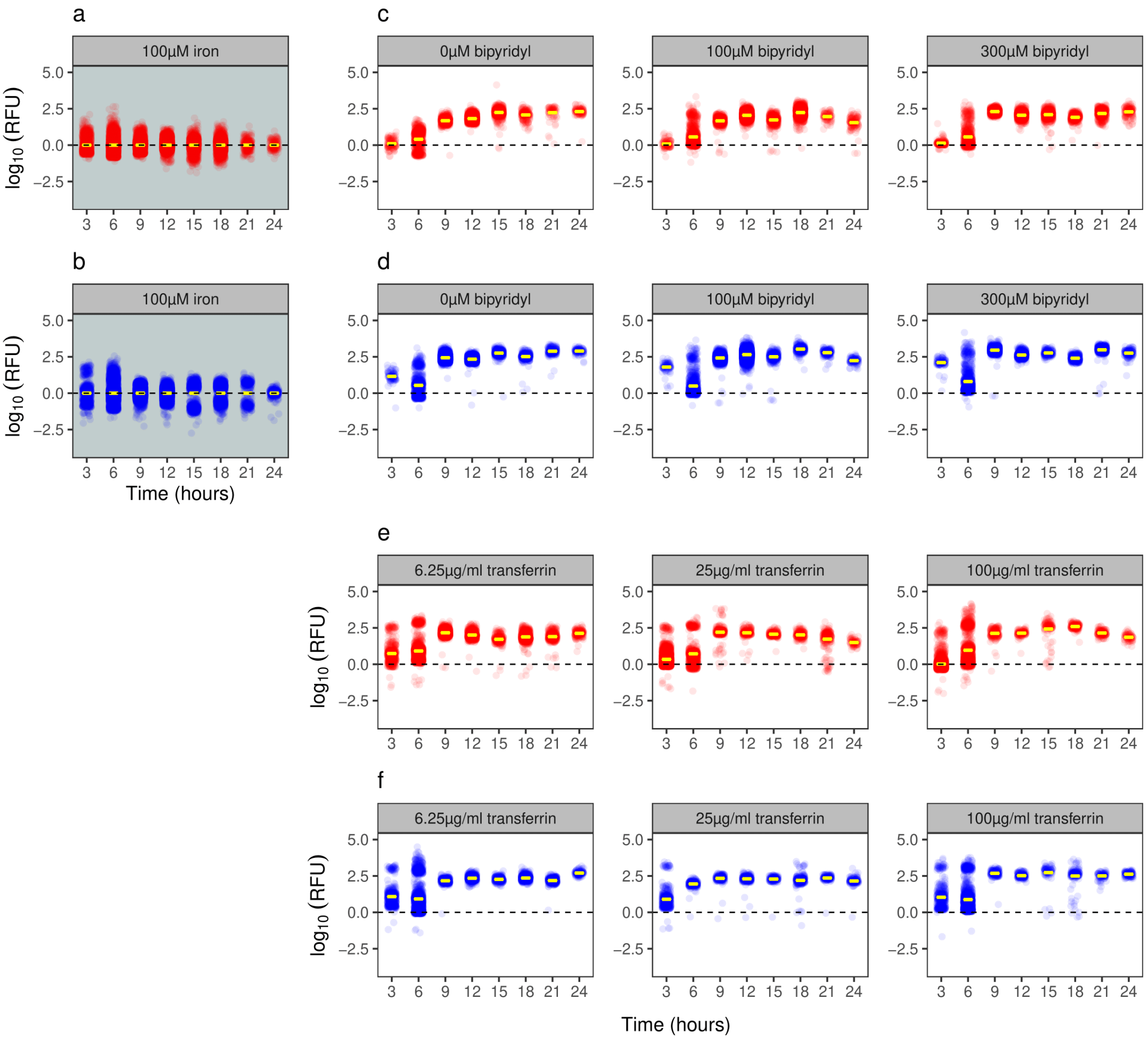

Figure S7. Single-cell siderophore gene expression patterns across time and media. The expression of pyochelin (red: *pchEF*) and pyoverdine (blue: *pvdA*) synthesis genes measured with the single gene reporters PAO1*pchEF*:*mcherry* and PAO1*pvdA*:*mcherry*, respectively across a range of CAA media differing in their levels of iron limitations. Each dot represents an individual cell with gene expression shown as log-transformed fluorescence values. (**a**+**b**) Iron-replete CAA medium (100 µM $FeCl_3$); (**c**+**d**) CAA media with increasing concentration of the iron chelator bipyridyl; (**e**+**f**) CAA media with increasing concentration of the iron chelator apo-transferrin. Relative fluorescence units (RFU) of the reporter proteins represent the gene expression intensity of single cells. Yellow bars represent the mean gene expression of all cells at a specific timepoint. The Pearson correlation coefficient r and the p-value of gene expression from the 9th hour onwards are provided in each panel.

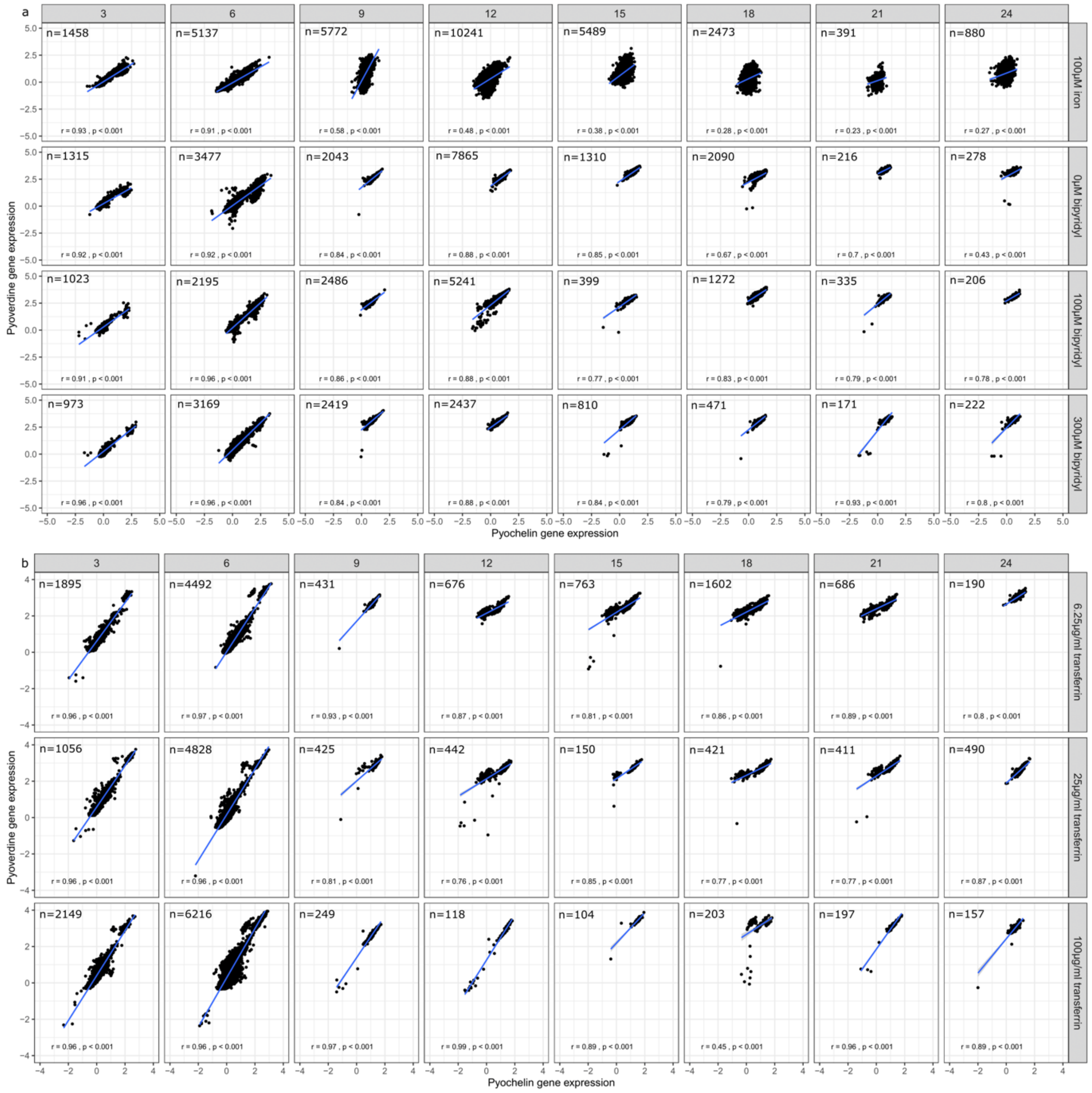

Figure S8. Correlations between pyochelin and pyoverdine gene expression across individual cells over time and in media differing in their level of iron limitation. All correlations between pyochelin and pyoverdine gene expression are measured with the double reporter strain PAO1*pvdA*::*mcherry–pchEF*::*egfp*. Each dot represents a single cell with its corresponding pyochelin (*pchEF)* and pyoverdine (*pvdA)* gene expression. Gene expression was measured every three hours (from left to right) by analysing a subset of cells extracted from growing cultures. Rows depict the different media conditions. **(a)** Gene expression correlations in iron-supplemented CAA medium (top row) and in CAA media supplemented with increasing concentration of the iron chelator bipyridyl (top-down). **(b)** Gene expression correlations in CAA media supplemented with increasing concentrations of the iron chelator apo-transferrin (top-down). The Pearson correlation coefficient r, the p-value and the sample size n are provided in each panel.

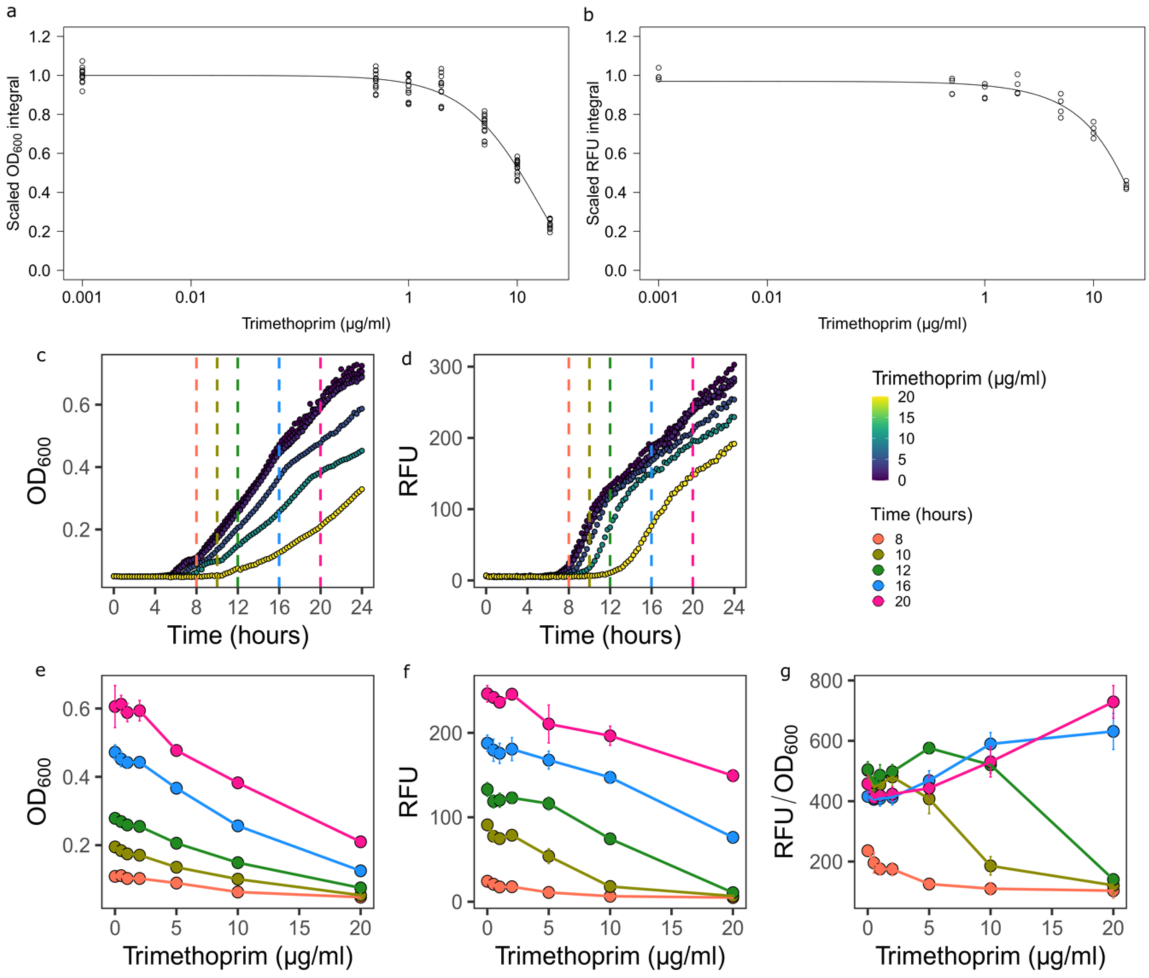

Figure S9. Effect of metabolic inhibitor trimethoprim on growth and *rpsL* gene expression. The following strains: PAO1 wildtype, PAO1*pchEF*:*mcherry,* PAO1*pvdA:mcherry* and PAO1*rpsL*:*mcherry* were subjected to varying concentration of trimethoprim in three-fold replication for the completeness of our experimental design. Trimethoprim inhibited the growth of all four strains in the same way, therefore we combined the growth data from all strains in panels **a, c** and **e**. In panels **b, d, f** and **g**, only data from the single reporter PAO1*rpsL*:*mcherry* were used. **(a+b)** Growth measured as integral (area under the OD600 curve) and *rpsL* gene expression measured as integral using the single reporter PAO1*rpsL*:*mcherry* (area under the fluorescence signal curve) over 24 hours batch culture experiments in CAA media with increasing concentrations of the metabolic inhibitor trimethoprim. The growth and *rpsL* gene expression integral were scaled (divided by integral values in CAA medium containing no trimethoprim) and represented as dose response curves. **(c)** Growth measured as OD600 and **(d)** *rpsL* gene expression measured as relative fluorescence units (RFU) across the 24 hours growth cycle at different concentration of trimethoprim. **(e)** Growth, **(f)** *rpsL* gene expression and **(g)** per capita *rpsL* gene expression at specific timepoints with increasing concentration of trimethoprim. **(g)** The results show that trimethoprim reduces per capita *rpsL* gene expression early on during the treatment, up to 12 hours, in a dose-dependent manner. At later time points, from 16 hours onwards, the impact of trimethoprim on metabolism wanes. This suggests that cells surviving the treatment reactivate their metabolism and increase overall gene expression, including *rpsL*. Values and error bars represent the mean and standard deviation across 12 replicates for growth and 3 replicates for *rpsL* gene expression.

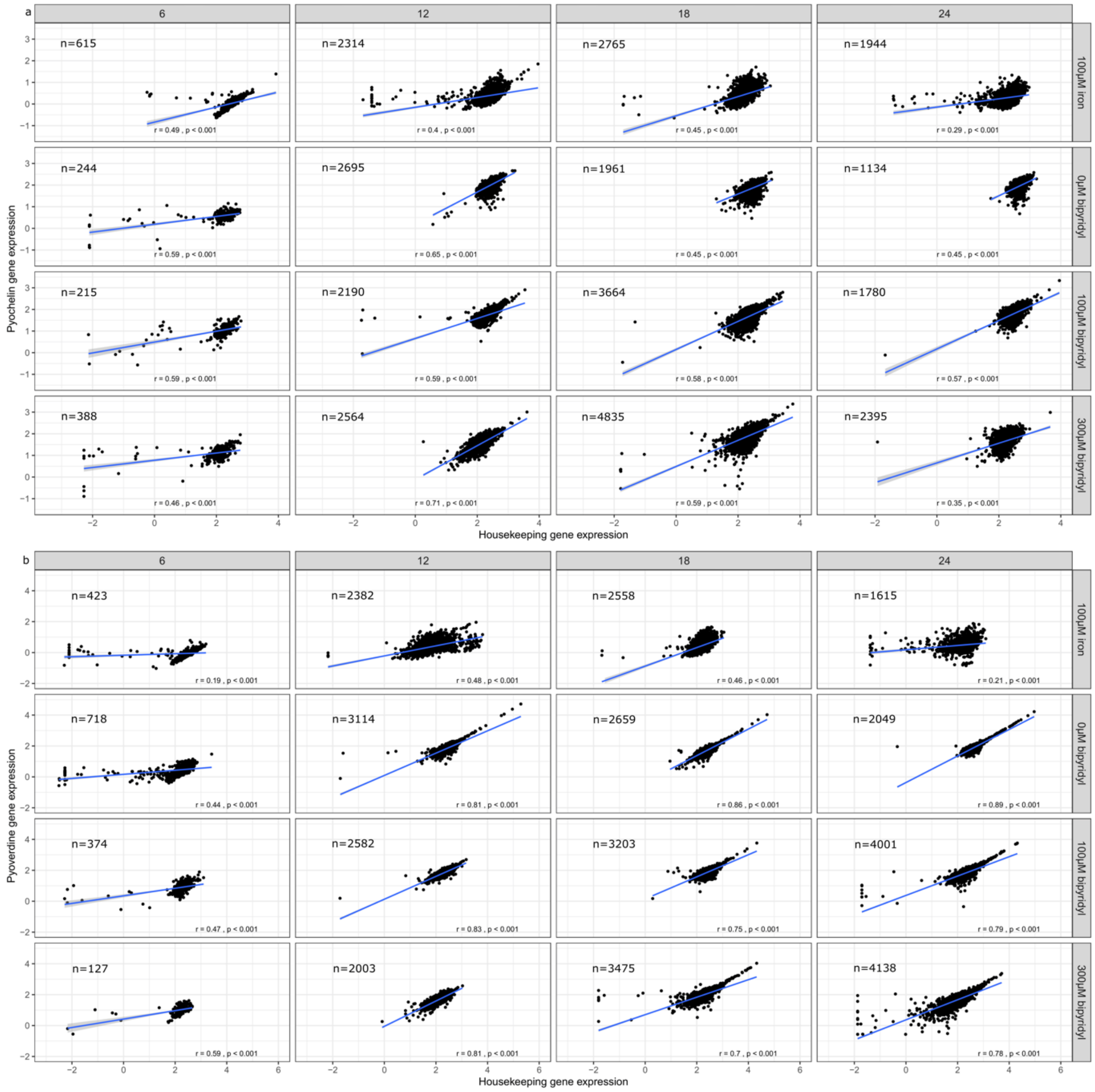

Figure S10. Correlations between siderophore and *rpsL* housekeeping gene expression across individual cells over time and in media differing in their level of iron limitation. (a) Correlations between pyochelin and housekeeping gene expression are measured with the double reporter strain PAO1*pchEF*::*mcherry–rpsL*::*egfp* . **(b)** Correlations between pyoverdine and housekeeping gene expression are measured with the double reporter strain PAO1*pvdA*::*mcherry–rpsL*::*egfp*. For both strains, gene expression was measured every six hours (from left to right) by analysing a subset of cells extracted from growing cultures. Rows depict the different media conditions: iron-supplemented CAA medium (top row) and CAA media supplemented with increasing concentrations of the iron chelator bipyridyl. Each dot represents a single cell. The Pearson correlation coefficient r, the p-value and the sample size n are provided in each panel.

Table S1: Comparison of growth parameters of *P. aeruginosa* populations across a range of casamino acids medium (CAA) compositions.

CAA supplement	concentration	growth integral	growth rate (ΔOD600/h)	lag phase (h)
FeCl3 (μM)	100	12.296 ± 0.153	0.273 ± 0.005	8.087 ± 0.030
No supplement	na	4.512 ± 0.027	0.089 ± 0.010	6.761 ± 0.030
Bipyridyl (μM)	50	4.946 ± 0.084	0.132 ± 0.067	6.784 ± 0.134
Bipyridyl (μM)	100	4.993 ± 0.079	0.064 ± 0.001	7.200 ± 0.098
Bipyridyl (μM)	150	4.868 ± 0.066	0.101 ± 0.035	7.425 ± 0.112
Bipyridyl (μM)	200	4.623 ± 0.090	0.073 ± 0.006	7.492 ± 0.354
Bipyridyl (μM)	300	3.557 ± 0.116	0.051 ± 0.001	9.016 ± 0.154
Transferrin (μg/ml)	6.25	4.095 ± 0.074	0.082 ± 0.023	5.077 ± 0.401
Transferrin (μg/ml)	12.5	1.319 ± 0.043	0.015 ± 0.001	11.029 ± 0.696
Transferrin (μg/ml)	25	0.960 ± 0.021	0.017 ± 0.003	12.453 ± 0.716
Transferrin (μg/ml)	50	0.876 ± 0.055	0.016 ± 0.001	13.743 ± 0.756
Transferrin (μg/ml)	100	0.952 ± 0.028	0.015 ± 0.001	12.940 ± 0.605

To estimate growth parameters, we fitted logistic growth models to our kinetic data (optical density at 600 nm) collected with the pate reader (Tecan, Männedorf, Switzerland). While the extracted values are useful for comparisons across treatments, it is important to note that the lag-phase values might be slightly overestimated because the plate reader is not sensitive enough to measure the early onset of growth.

Table S2: List of strains

**Strain name**	**Description or genotype**	**Source or reference**
E. coli		
CC118 λpir	Δ (*ara, leu*)$_{7697}$ *araD*139 *ΔlacX74 galE galK phoA20 thi-1 rpsE rpoB* (Rf^R) *argE(am) recA1 λpir*$^+$	De Lorenzo *et al.*, 1990(1)
P. aeruginosa		
PAO1 (ATCC 15692)	Wild type strain	This laboratory
***P. aeruginosa* PAO1 (single fluorescent gene reporters)**		
PAO1*pvdA:mcherry*	Transcriptional fusion *pvdA::mcherry* from pSR01	Rezzoagli *et al.* 2019(2)
PAO1*pchEF:mcherry*	Transcriptional fusion *pchEF::mcherry* from pSR02	Rezzoagli *et al.* 2019(2)
PAO1*rpsL:*mcherry	Transcriptional fusion *rpsL*::mcherry from pSR03	Jayakumar *et al.* (unpublished, this laboratory)
***P. aeruginosa* PAO1 (double fluorescent gene reporters)**		
PAO1*pvdA*: mcherry-*pchEF*::GFP	Transcriptional fusion *pvdA*::mcherry and *pchEF*::GFP from pDR01	This study
PAO1*pvdA*: mcherry-*rpsL*::GFP	Transcriptional fusion *pvdA*::mcherry and *rpsL*::GFP from pDR02	This study
PAO1*pchEF*: mcherry-*rpsL*::GFP	Transcriptional fusion *pchEF*::mcherry and *rpsL*::GFP from pDR03	This study

Table S3: List of plasmids

Plasmid name	Description or genotype	Source or reference
pEX-A128-*pvdA::mcherry*	Commercial plasmid with *pchEF::mcherry* between HindIII/SacI sites	Weigert *et al*., 2017(3)
pUX-BF13	Helper plasmid to provide Tn7 transposase proteins	Bao *et al*., 1991(4)
pUC18-miniTn7-Gm	Gm^r on mini-Tn7; for chromosomal insertion in Gm^s bacteria in the *att*Tn7 site	Choi and Schweizer *et al*., 2006(5)
pUC18-miniTn7-Gm-mcherry-GFP	Derived from pUC18-miniTn7-Gm-mcherry; with amplified GFP from pEX-A128-*pchEF*::GFP	This study
pDR01	pUC18-mini-Tn7-Gm with *pvdA*::mcherry and *pchEF*::GFP	This study
pDR02	pUC18-mini-Tn7-Gm with *pvdA*::mcherry and *rpsL*::GFP	This study
pDR03	pUC18-mini-Tn7-Gm with *pchEF*::mcherry and *rpsL*::GFP	This study

Table S4: List of primers

Primer name	Sequence (5'-3')	Application	Template
pvdA_Rev_Sac1	CGG CAT CAG AGC AGA TTG TA	Plasmid pDR01	pEX-pvdA-*mcherry*
pvdA_Fwd_HindIII	GGA TCC AAG CGA GCA AAA G	Plasmid pDR01	pEX-pvdA-*mcherry*
pchEF_Fwd_HindIII	GATCAA GCTTCAAGCGCTACG GCATCTC	Plasmid pDR01 & pDR03	PAO1 gDNA
pchEF_Rev_PacI	CGA G TTAATTAA TC ACT GCT CGG TCA GCC AGT C	Plasmid pDR01	PAO1 gDNA
pvdA_Fwd_HindIII_2	CAGTGCAGGTGGGAAGCTTATGC	Plasmid pDR02	pEX-pvdA-*mcherry*
pvdA_Rev_BamHI	CAGTCCTCCTTCTTAAAGGATCC	Plasmid pDR02	pEX-pvdA-*mcherry*
rpsL_Fwd_HindIII	CAGTAAGCTTGTACCGGTCTGGCTTAC CAC	Plasmid pDR02 & pDR03	PAO1 gDNA
rpsL_Rev_BamHI	CAGTGGATCCTCAGTGTGCCGAGTTCG GCTTTT	Plasmid pDR02 & pDR03	PAO1 gDNA
pchEF_Rev_BamHI	CGA GGGATCCTC ACTGCTCGGTCAGCCAGT C	Plasmid pDR03	PAO1 gDNA
Fwd_HindIII_Tn7 (for pDR02)	GCGCGAATGGGAAGCCGACTG	*E. coli* colonies	Colony PCR to check insertion in *E. coli*
Fwd_HindIII_pEx (pDR03)	GAT CAA GCT TCA AGC GCT ACG GCA TCT C	*E. coli* colonies	Colony PCR to check

			insertion in *E. coli*
Rev_Tn7_primer (for pDR02 & pDR03)	CGAACCGAACAGGCTTATGT	*E. coli* colonies	Colony PCR to check insertion in *E. coli*
mcherry_rev2	GGATATCCGCTGGGTGTTTA	*P. aeruginosa* colonies	Colony PCR to check insertion in *P. aeruginosa*

To confirm the insertion of the promoter region into the miniTn7 vector, colony PCR was performed using the mcherry_rev2 primer, together with the corresponding "Fwd_HindIII" promoter-specific primers

Supplementary References

1. V. De Lorenzo, M. Herrero, U. Jakubzik, K. N. Timmis, Mini-Tn5 transposon derivatives for insertion mutagenesis, promoter probing, and chromosomal insertion of cloned DNA in gram-negative eubacteria. *J. Bacteriol.* **172**, 6568–6572 (1990).
2. C. Rezzoagli, E. T. Granato, R. Kümmerli, In-vivo microscopy reveals the impact of *Pseudomonas aeruginosa* social interactions on host colonization. *ISME J.* (2019).
3. M. Weigert, R. Kümmerli, The physical boundaries of public goods cooperation between surface-attached bacterial cells. *Proc. R. Soc. B Biol. Sci.* **284** (2017).
4. Y. Bao, D. P. Lies, H. Fu, G. P. Roberts, An improved Tn7-based system for the single-copy insertion of cloned genes into chromosomes of gram-negative bacteria. *Gene* **109**, 167–168 (1991).
5. K. H. Choi, H. P. Schweizer, mini-Tn7 insertion in bacteria with single attTn7 sites: Example *Pseudomonas aeruginosa*. *Nat. Protoc.* **1**, 153–161 (2006).
