# Supplementary material for: Coordination of siderophore gene expression among clonal cells of the bacterium Pseudomonas aeruginosa
Source: Commun Biol. 2022 Jun 6;5:545. doi: 10.1038/s42003-022-03493-8 (PMC9170778; doi:10.1038/s42003-022-03493-8)
Supplement: Supplementary file 3 — Description of Additional Supplementary Files [file 42003_2022_3493_MOESM3_ESM.pdf]

Description of Additional Supplementary Files

File name: Supplementary Software 1
Description: Unpacks the microscope image files into different channels

File name: Supplementary Software 2
Description: Obtains single cell information from images of different channels
