# Supplementary material for: Coordination of siderophore gene expression among clonal cells of the bacterium Pseudomonas aeruginosa
Source: Commun Biol. 2022 Jun 6;5:545. doi: 10.1038/s42003-022-03493-8 (PMC9170778; doi:10.1038/s42003-022-03493-8)
Supplement: Supplementary file 6 — Reporting Summary [file 42003_2022_3493_MOESM6_ESM.pdf]

nature research

Corresponding author(s): Subham Mridha & Rolf Kümmerli

Last updated by author(s): Apr 25, 2022

Reporting Summary

Nature Research wishes to improve the reproducibility of the work that we publish. This form provides structure for consistency and transparency in reporting. For further information on Nature Research policies, see our Editorial Policies and the Editorial Policy Checklist.

Statistics

For all statistical analyses, confirm that the following items are present in the figure legend, table legend, main text, or Methods section.

n/a	Confirmed	
☐	☒	The exact sample size (n) for each experimental group/condition, given as a discrete number and unit of measurement
☐	☒	A statement on whether measurements were taken from distinct samples or whether the same sample was measured repeatedly
☐	☒	The statistical test(s) used AND whether they are one- or two-sided<br>*Only common tests should be described solely by name; describe more complex techniques in the Methods section.*
☐	☒	A description of all covariates tested
☐	☒	A description of any assumptions or corrections, such as tests of normality and adjustment for multiple comparisons
☐	☒	A full description of the statistical parameters including central tendency (e.g. means) or other basic estimates (e.g. regression coefficient) AND variation (e.g. standard deviation) or associated estimates of uncertainty (e.g. confidence intervals)
☐	☒	For null hypothesis testing, the test statistic (e.g. F, t, r) with confidence intervals, effect sizes, degrees of freedom and P value noted<br>*Give P values as exact values whenever suitable.*
☒	☐	For Bayesian analysis, information on the choice of priors and Markov chain Monte Carlo settings
☒	☐	For hierarchical and complex designs, identification of the appropriate level for tests and full reporting of outcomes
☐	☒	Estimates of effect sizes (e.g. Cohen's d, Pearson's r), indicating how they were calculated

Our web collection on statistics for biologists contains articles on many of the points above.

Software and code

Policy information about availability of computer code

Data collection	We used the open source statistical programming language R (ver 3.4.2) to curate our data<br>We used the open source machine learning software Ilastik (ver 1.3.0) to classify objects (distinguish single bacterial cells from background,in phase contrast microscopy images obtained using fluorescence microscope)<br>We used the open source software FIJI to extract informations from single cells (like fluorescence, area) by using codes (macros) previously described by<br>Weigert, M. & Kümmerli, R. The physical boundaries of public goods cooperation between surface attached bacterial cells. Proc. R. Soc. B 284, 20170631, (2017)<br>We are uploading the modified codes used in our manuscript as Supplementary Software files along with manuscript.
Data analysis	We used the open source statistical programming language R (ver 3.4.2) to perform data analysis

For manuscripts utilizing custom algorithms or software that are central to the research but not yet described in published literature, software must be made available to editors and reviewers. We strongly encourage code deposition in a community repository (e.g. GitHub). See the Nature Research guidelines for submitting code & software for further information.

Data

Policy information about availability of data

All manuscripts must include a data availability statement. This statement should provide the following information, where applicable:

- Accession codes, unique identifiers, or web links for publicly available datasets
- A list of figures that have associated raw data
- A description of any restrictions on data availability

All the data sets generated during the current study will be made available in the figshare repository upon acceptance of this manuscript for publication.

April 2020

Field-specific reporting

Please select the one below that is the best fit for your research. If you are not sure, read the appropriate sections before making your selection.

☐ Life sciences ☐ Behavioural & social sciences ☒ Ecological, evolutionary & environmental sciences

For a reference copy of the document with all sections, see nature.com/documents/nr-reporting-summary-flat.pdf

Ecological, evolutionary & environmental sciences study design

All studies must disclose on these points even when the disclosure is negative.

Study description	Our study focuses on the coordination of gene expression in the bacterium Pseudomonas aeruginosa at population and single cell level. We used fluorescent reporter strains to study the siderophore genes pyoverdine and pyochelin, using wide-field fluorescence microscopy.
Research sample	We used the model organism Pseudomonas aeruginosa PAO1 (ATCC 15692) and fluorescence reporter variants made in this strain background using the protocol described by<br>Choi, K.-H. & Schweizer, H. P. mini-Tn7 insertion in bacteria with single attTn7 sites: example Pseudomonas aeruginosa. Nat. Protoc. 1, 153-161, (2006).
Sampling strategy	We imaged Pseudomonas aeruginosa cells on a solid agarose patch. Images were taken with a fluorescence microscope, for fields of views with individually discernible cells. In total 327 113 cells were sampled and quantified.
Data collection	Images were taken with a fluorescence microscope, sample phase contrast images were used to train the machine learning software Ilastik to segment the images (distinguish bacterial cells from background). After segmenting all acquired images using the Ilastik training protocol, the corresponding segmented images (binary images) were used to obtain fluorescence data from original images in the respective fluorescent channels (mcherry, GFP). The data collection and curation was done by Subham Mridha
Timing and spatial scale	Data was collected over a period of 24 hours for each experiment. Images of bacteria growing in a cell cultures was obtained using a microscope every 3 hours.
Data exclusions	Segmentation with Ilastik led to artifacts like classification of non bacterial cells (objects) within phase contrast images and the resulting binary images. Therefore we set a cut off for the bacterial cell size and every cell (object) above that cut off were classified as true cells and objects. Data analysis were performed after this step.
Reproducibility	Each experiments were performed 2 to 4 times independently.
Randomization	The first part of our study deals with population level analysis, where bacteria were grown in 96 well plates. Each treatment was replicated at least thrice and their position in the 96 well plate was random. The second part of our study deals with gene expression in single cells across different media over time using microscope. The choice of the field of view for imaging was random. The only criteria that the field of view had to contain individually discernible cells.
Blinding	Blinding was not necessary for our study as all analysis were based on fully automated machines and analysis. No manual counting or measurements were involved that could lead to biases. The microscopy experiments were insofar blinded that the fields of view were chosen based on neutral phase-contrast images. Thus, the choices were blind in relation to the fluorescence signals, which were quantified afterwards.

Did the study involve field work? ☐ Yes ☒ No

Reporting for specific materials, systems and methods

We require information from authors about some types of materials, experimental systems and methods used in many studies. Here, indicate whether each material, system or method listed is relevant to your study. If you are not sure if a list item applies to your research, read the appropriate section before selecting a response.

Materials & experimental systems

n/a	Involved in the study	
☒	☐	Antibodies
☒	☐	Eukaryotic cell lines
☒	☐	Palaeontology and archaeology
☐	☒	Animals and other organisms
☒	☐	Human research participants
☒	☐	Clinical data
☒	☐	Dual use research of concern

Methods

n/a	Involved in the study	
☒	☐	ChIP-seq
☒	☐	Flow cytometry
☒	☐	MRI-based neuroimaging

Animals and other organisms

Policy information about studies involving animals; ARRIVE guidelines recommended for reporting animal research

Laboratory animals	Pseudomonas aeruginosa PAO1 (ATCC 15692)
Wild animals	Study did not involve wild animals
Field-collected samples	Study did not involve sample collected from field
Ethics oversight	There is no ethical guidance required for our study. The study deals with the bio-safety level 2 organism Pseudomonas aeruginosa PAO1. Our research lab has the relevant authorisation to work with this pathogen.

Note that full information on the approval of the study protocol must also be provided in the manuscript.
